# Docosahexaenoic Acid-Infused Core–Shell Fibrous Membranes for Prevention of Epidural Adhesions

**DOI:** 10.3390/ijms252313012

**Published:** 2024-12-03

**Authors:** Zhuo-Hao Liu, Yin-Cheng Huang, Chang-Yi Kuo, Darshan Tagadur Govindaraju, Nan-Yu Chen, Ping K. Yip, Jyh-Ping Chen

**Affiliations:** 1 Department of Neurosurgery, Chang Gung Memorial Hospital, Linkou, Chang Gung University School of Medicine, Kwei-San, Taoyuan 33305, Taiwan; b8402022@gmail.com (Z.-H.L.);; 2 Department of Chemical and Materials Engineering, Chang Gung University, Kwei-San, Taoyuan 33302, Taiwan; 3 Department of Internal Medicine, Chang Gung Memorial Hospital, Linkou, Chang Gung University School of Medicine, Kwei-San, Taoyuan 33305, Taiwan; 4 Centre for Neuroscience, Surgery & Trauma, Blizard Institute, Barts and the London School of Medicine and Dentistry, Queen Mary University of London, London E1 2AT, UK; 5 Research Center for Food and Cosmetic Safety, College of Human Ecology, Chang Gung University of Science and Technology, Taoyuan 33305, Taiwan; 6 Department of Materials Engineering, Ming Chi University of Technology, Tai-Shan, New Taipei City 24301, Taiwan

**Keywords:** adhesion, docosahexaenoic acid, electrospinning, fibers, membranes, polylactic acid

## Abstract

Avoiding epidural adhesion following spinal surgery can reduce clinical discomfort and complications. As the severity of epidural adhesion is positively correlated with the inflammatory response, implanting a fibrous membrane after spinal surgery, which can act as a physical barrier to prevent adhesion formation while simultaneously modulates postoperative inflammation, is a promising approach to meet clinical needs. Toward this end, we fabricated an electrospun core–shell fibrous membrane (CSFM) based on polylactic acid (PLA) and infused the fiber core region with the potent natural anti-inflammatory compound docosahexaenoic acid (DHA). The PLA/DHA CSFM can continuously deliver DHA for up to 36 days in vitro and reduce the penetration and attachment of fibroblasts. The released DHA can downregulate the gene expression of inflammatory markers (IL-6, IL-1β, and TNF-α) in fibroblasts. Following an in vivo study that implanted a CSFM in rats subjected to lumbar laminectomy, the von Frey withdrawal test indicates the PLA/DHA CSFM treatment can successfully alleviate neuropathic pain-like behaviors in the treated rats, showing 3.60 ± 0.49 g threshold weight in comparison with 1.80 ± 0.75 g for the PLA CSFM treatment and 0.57 ± 0.37 g for the untreated control on day 21 post-implantation. The histological analysis also indicates that the PLA/DHA CSFM can significantly reduce proinflammatory cytokine (TNF-α and IL-1β) protein expression at the lesion and provide anti-adhesion effects, indicating its vital role in preventing epidural fibrosis by mitigating the inflammatory response.

## 1. Introduction

It has been reported that 8–40% of patients experience failed back surgery syndrome (FBSS), and 4–9% of patients receive secondary surgical intervention following spinal surgery [1]. The migration of fibroblasts and inflammatory factors from the surgical site leads to the formation of epidural fibrosis at the surgical area, which is a natural healing response after spinal surgery. However, severe adhesion to the dura mater from the epidural fibrosis causes intractable leg and back pain, which is the major cause of FBSS [2]. The pathophysiological event contributing to post-operative epidural fibrosis is associated with fibroblast proliferation and infiltration, upregulation of proinflammatory cytokines, and hematoma formation following surgery [3]. Although the pathological mechanism responsible for the development of FBSS is still under investigation, migrated paravertebral fibroblasts can lead to epidural fibrosis and be considered a major pathophysiological event responsible for FBSS.

Membranes made from biocompatible and biodegradable materials can be used as physical barriers to reduce post-operative adhesion formation [4]. Specifically, antifibrotic membranes can isolate the infiltration of fibrotic factors to minimize epidural adhesion for FBSS relief [5,6]. However, the inflammatory response stimulated by foreign body reactions may overwhelm the advantages of such physical barriers [7]. The pathological mechanisms of FBSS may be linked to inflammation-related arachnoiditis, which is a potential cause of post-operative neurological deficits [8]. Additionally, a study by Lee et al. indicated that reducing inflammation can lead to decreased neurologic deficits following spinal surgery [9]. This suggests that addressing inflammation is an important approach to managing FBSS. Other than mitigating the inflammatory response, amniotic membranes can reduce epidural fibrosis after laminectomy by reducing epidural scar adhesion [10]. However, the removal of these barriers by macrophages during the biodegradation process, combined with the inflammation and cytokine release, can trigger a foreign body reaction and subsequently reduce the anti-adhesion efficacy [11,12]. Therefore, it is essential to use an anti-adhesion physical barrier membrane that can effectively inhibit acute and secondary inflammation to ameliorate both epidural fibrosis and arachnoiditis after spinal surgery.

With the rapid development of biomaterial science and multidisciplinary research, biodegradable electrospun fibrous membranes have attracted huge interest as anti-adhesion biomaterials that act as a physical barrier to prevent adhesion formation [13]. These membranes are characterized by a fibrous microstructure with interconnected pores and high porosity [14]. They can provide a flexible membrane structure with controllable pore size to facilitate nutrient transport and promote intrinsic healing, while preventing the attachment and penetration of fibroblasts responsible for adhesion formation [15]. Using a spinneret with an inner core and outer shell channels, co-axial electrospinning can produce fibrous membranes composed of nano- to micro-size core–shell fibers. These membranes can provide exquisite physicochemical properties not found in other materials [16]. The core–shell structure can accommodate a bioactive molecule or drug in the core region of the fiber and allow its sustained release to exert biological functions [17]. Specifically, an electrospun core–shell fibrous membrane (CSFM) could be used for the controlled release of an anti-inflammatory drug to reduce adhesion formation following surgery [18]. Furthermore, human lumbar dura fibers are typically aligned longitudinally, which gives them anisotropic mechanical properties [19,20]. Therefore, aligned arrays of electrospun fibers could be an ideal substitute for anti-adhesion materials when loaded with a therapeutic compound.

Docosahexaenoic acid (DHA) is one of the polyunsaturated fatty acids that can influence the inflammatory response through many mechanisms [21]. Compared to other anti-inflammatory agents, such as non-steroidal anti-inflammatory drugs (NSAIDs) and steroids, which can be cytotoxic to local tissues or the nervous system at high concentrations, DHA is a natural compound that offers neuroprotective and anesthetic effects. Several clinical and experimental studies indicated that DHA could be a novel and highly effective agent for minimizing adhesive disease [22,23,24,25]. A recent study also showed that DHA could largely abolish fibroblast activation and inflammatory cell accumulation [26]. DHA also significantly reduces the expression levels of certain adhesion markers in fibroblasts to a normal or below-normal level compared to untreated fibroblasts [23]. Polylactic acid (PLA) is a popular biocompatible, biodegradable, nontoxic polymer suitable for many biomedical applications [27]. Therefore, we aim to develop an electrospun PLA/DHA CSFM that can reduce spinal scar formation, inhibit the inflammatory process, and reduce fibroblast attachment and penetration in this study. The physical properties of the membrane, drug release profile, in vitro cellular response toward fibroblasts, and in vivo anti-adhesion effects were investigated. Overall, the PLA/DHA CSFM was found to have great potential to prevent the formation of epidural adhesions.

## 2. Results

### 2.1. Characterization of Core-Shell Fibrous Membrane (CSFM)

**Figure 1:**
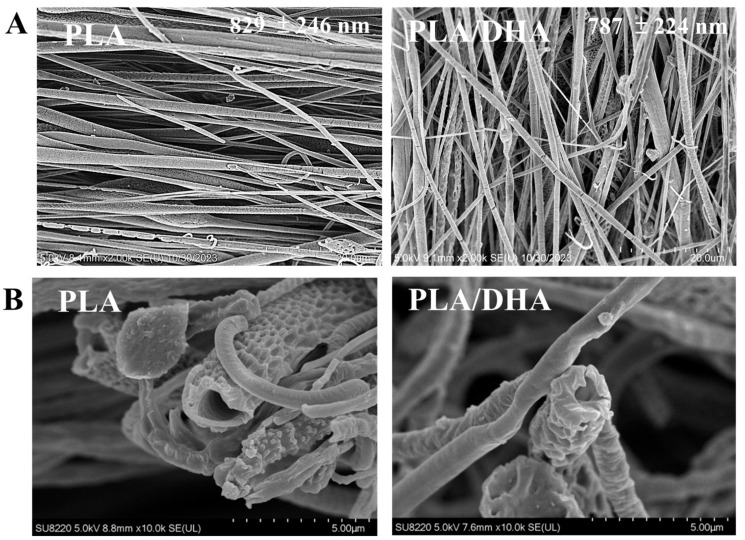
The field-emission scanning electron microscope (FE-SEM) analysis of a PLA and a PLA/DHA core–shell fibrous membrane (CSFM) ((**A**), bar = 20 μm), and the membranes after soaking in distilled water for 3 days ((**B**), bar = 5 μm).

The morphology of the CSFMs was examined using a field-emission scanning electron microscope (FE-SEM), and a dense array of well-arranged fibers is evident, as shown in Figure 1A. The average fiber diameter was estimated to be 829 ± 246 nm for PLA and 787 ± 224 nm for PLA/DHA, with no statistical difference between them. The core–shell structure of both CSFMs can be easily observed after breaking a membrane in liquid nitrogen and examining its cross section with FE-SEM (Figure 1B).

**Figure 2:**
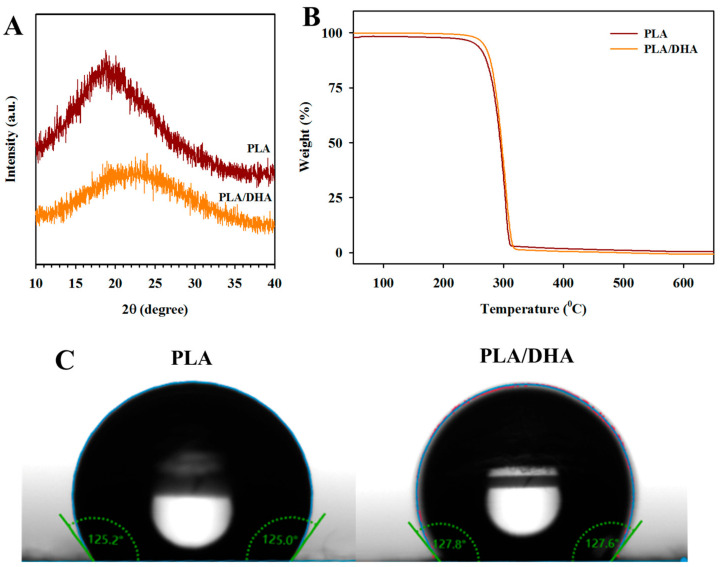
The X-ray diffraction (XRD) analysis (**A**), the thermal gravimetric analysis (TGA) (**B**), and the water contact angles (**C**) of a PLA and a PLA/DHA core–shell fibrous membrane (CSFM).

From the X-ray diffraction (XRD) analysis, the PLA CSFM displays a semi-crystalline peak at 2θ = 18.9°, arising from the fast evaporation of the solvent during the formation of fibers in the electrospinning process, which restricts the mobility of the polymeric chains and leads to reduced crystallinity (Figure 2A). There is a slight shift of the peak to a higher 2θ value (22.0°) and the peak becomes broader for PLA/DHA, indicating that DHA may be carried by the evaporated ethanol into the shell to interfere with PLA crystallization. The blending of DHA with PLA may reduce the crystallinity of the PLA/DHA CSFM. This was further confirmed by the TGA, where PLA/DHA shows higher heat resistance than PLA [28]. The 5% thermal weight loss temperature increased from 253 °C for PLA to 268 °C for PLA/DHA, while the 10% thermal weight loss increased from 267 °C to 275 °C (Figure 2B). The water contact angle analysis indicated a slight increase in the value of the water contact angle or the hydrophobicity of the membrane due to residual DHA on the fiber surface (Figure 2C). Taken together, the presence of DHA in the PLA/DHA electrospun fibers is confirmed.

**Figure 3:**
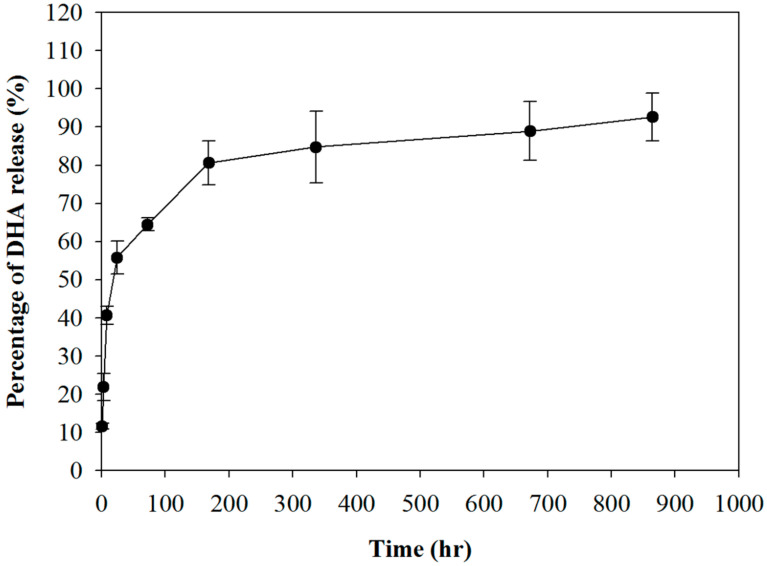
The release profiles of DHA from a PLA/DHA core–shell fibrous membrane (CSFM) in phosphate-buffered saline (PBS) at 37 °C.

The kinetics of DHA release from the PLA/DHA CSFM was studied by immersing 100 mg of the fibrous membrane in 1 mL of PBS at 37 °C. The amount of DHA released was determined using an enzyme-linked immunosorbent assay (ELISA) kit to calculate the release percentage of DHA based on the amount of DHA used for preparing the membrane. As shown in Figure 3, DHA exhibited a two-stage drug release pattern, with an initial burst release within 24 h, followed by a sustained release behavior of up to 36 days. The percentage of cumulative rerelease of DHA reached 55.8% within 24 h, and the release of DHA could be extended to 92.6% at the end of the experimental period (36 days).

### 2.2. In Vitro Cell Culture

**Figure 4:**
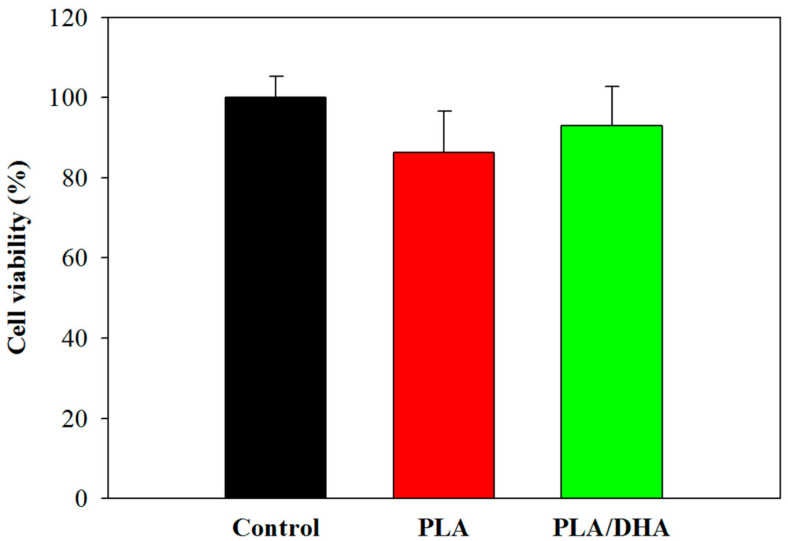
The cytotoxicity test of a PLA and a PLA/DHA core–shell fibrous membrane (CSFM) after culturing fibroblasts with the 24 h extraction medium of the PLA or PLA//DHA CSFM. The control group is fibroblasts cultured in a cell culture medium, which was taken as 100%.

Using a 24 h extract of a CSFM for cell culture, the potential cytotoxicity of the CSFM was assessed in accordance with ISO 10993-5 standards. The cell viability was assessed using 3-(4,5-Dimethylthiazol-2-yl)-2,5-diphenyltetrazolium bromide (MTT) assays by culturing fibroblasts with the extract for 24 h. The high biocompatibility of the PLA and PLA/DHA is shown in Figure 4, where no significant difference could be found between the samples and the control for both membranes (*p* > 0.05).

**Figure 5:**
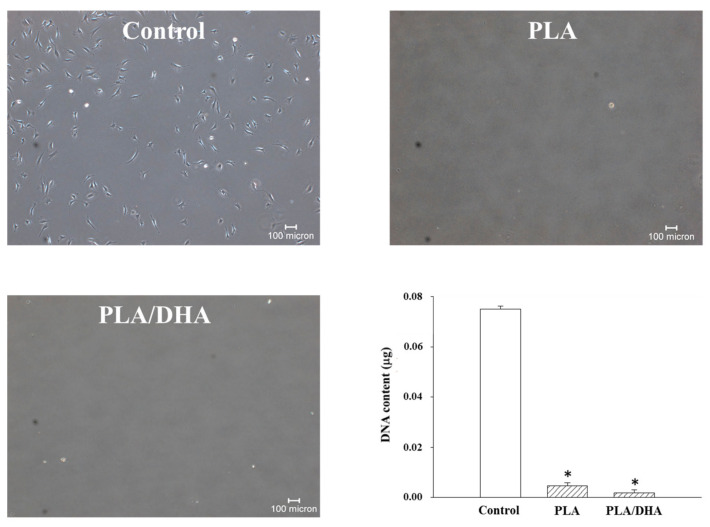
Penetration of 3T3 fibroblasts through a PLA or PLA/DHA core–shell fibrous membrane (CSFM) in 24 h based on microscopic observation and DNA assays. The control involved not placing a membrane in the cell insert during the cell penetration study. Bar = 100 μm. * *p* < 0.05 compared with control.

Adhesion formation is usually associated with the excessive deposition of the extracellular matrix (ECM) that is mainly synthesized by fibroblasts, making fibroblasts play a key role in epidural adhesion after laminectomy [29]. The barrier effect of the CSFMs in preventing adhesion formation was studied by placing fibroblasts on top of a CSFM in a cell insert. Cell penetration through the membrane, driven by an FBS gradient from 2 to 10% within 24 h, was studied through direct observation of the cell number in the bottom well with an inverted microscope and from DNA assays. Figure 5 shows that only a few fibroblasts can penetrate through the PLA or PLA/DHA CSFM and attach to the bottom of the well, in contrast to the abundant cells found in the control group without using a CSFM. The quantitative analysis of cell numbers from DNA analysis also showed a significantly lower DNA content for the PLA and PLA/DHA CSFM groups when compared with the control (without using a CSFM). The highly dense thick fibrous structure, small pore size, and narrow pore-size distribution of the CSFM can provide a cell barrier effect to prevent the penetration of adhesion-causing fibroblasts through the membrane and is expected to reduce post-surgical adhesion in vivo.

**Figure 6:**
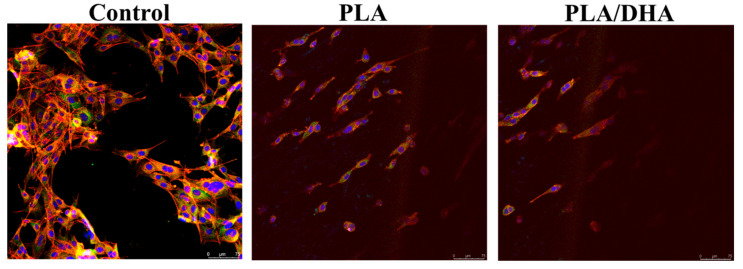
The immunofluorescence staining of fibroblasts cultured on tissue culture polystyrene (TCPS) (control) and a PLA or a PLA/DHA core–shell fibrous membrane (CSFM). Actin cytoskeleton arrangement (red) and the expression of the focal adhesion protein vinculin (green) on day 3 were examined under a laser confocal microscope after counterstaining the nuclei (blue). Bar = 75 μm.

Vinculin is an actin-binding protein and adhesion marker protein presented in the ECM. It plays an important role in regulating integrin clustering, as well as cell adhesion and movement, and is frequently used as a marker of focal adhesion [30]. To investigate the effect of DHA on reducing the attachment of fibroblasts to a CSFM, the morphology, cytoskeletal actin distribution, and expression of focal adhesion protein (vinculin) by fibroblasts were monitored for cells cultured on tissue culture polystyrene (TCPS) and the CSFM. From the immunofluorescence staining, fibroblasts cultured on TCPS demonstrated increased cellular spreading, well-distributed fibrous F-actin cytoskeleton, and enhanced vinculin expression (Figure 6). In contrast, fibroblasts cultured on PLA or PLA/DHA showed a more rounded morphology, diffused F-actin cytoskeletal distribution, and minimal vinculin expression (Figure 6). Fibroblasts do not adhere or proliferate well on the surface of the PLA CSFM due to the hydrophobicity of this polymer [31]. Showing a similar effect, the downregulated vinculin expression of fibroblasts on the PLA/DHA surface indicates that this CSFM preserves such an anti-adhesion effect. However, it is expected to be augmented with an additional anti-inflammatory effect not found in PLA due to the released DHA.

**Figure 7:**
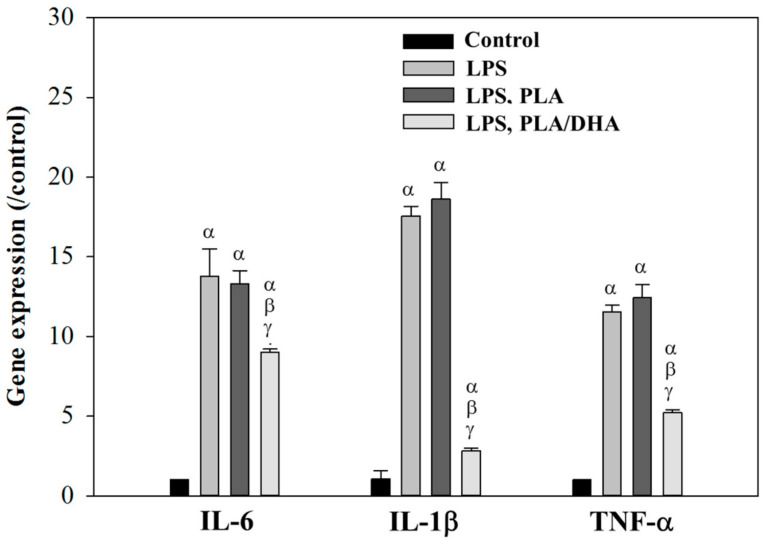
The mRNA expression of the proinflammatory cytokines interleukin-1β (IL-1β), interleukin-6 (IL-6), and tumor necrosis factor-α (TNF-α) in 3T3 fibroblasts based on reverse transcription polymerase chain reaction (RT-PCR). The cells were stimulated with 1 μg/mL LPS (LPS group), or with 1 μg/mL LPS and then cultured in the extract of a PLA (LPS, PLA group) or a PLA/DHA (LPS, PLA/DHA group) core–shell fibrous membrane (CSFM). The control group is 3T3 fibroblasts without LPS stimulation. ^α^ *p* < 0.05 compared with control, ^β^ *p* < 0.05 compared with LPS, and ^γ^ *p* < 0.05 compared with LPS, PLA.

Lipopolysaccharides (LPSs), which are components of the outer membrane of Gram-negative bacteria, are known to induce strong inflammatory responses. To test whether DHA-loaded CSFMs can reduce the inflammatory response in fibroblasts, we quantified the expression of genes involved in the inflammatory response after treating LPS-induced fibroblasts with the extracts of the CSFMs. After LPS stimulation, the gene expression of the proinflammatory cytokines interleukin-1β (IL-1β), interleukin-6 (IL-6), and tumor necrosis factor-α (TNF-α) in 3T3 fibroblasts was significantly upregulated compared to the control group without LPS induction (Figure 7). However, when treated with the extract of PLA or PLA/DHA, only the PLA/DHA group significantly reduced the expression levels of IL-6, IL-1β, and TNF-α genes (Figure 7). This suggests that the PLA/DHA CSFM can ameliorate the acute inflammatory response in 3T3 fibroblasts following surgical intervention.

### 2.3. Animal Study

**Figure 8:**
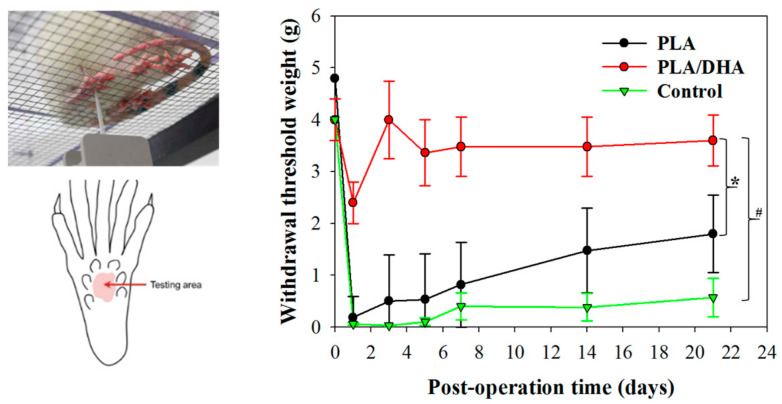
The mechanical allodynia test of rats before (day 0) and at different time points after lumbar laminectomy. The animals were subjected to spinal surgery (laminectomy) without treatment (control) or with treatment by implanting a PLA or a PLA/DHA core–shell fibrous membrane (CSFM) post-surgery. The von Frey test was used to determine the changes in the rats’ mechanical withdrawal threshold of the footpad in terms of the force applied. * *p* < 0.05 compared with PLA; ^#^ *p* < 0.05 compared with control.

To determine the mechanical hyperalgesia and tactile allodynia related to dura adhesion in rats after receiving surgery, the von Frey test was applied to animals receiving spinal surgery with or without CSFM implantation. There was no difference in the hind paw withdrawal thresholds at baseline on day 0 (before surgery) between the groups. The rats in the PLA/DHA group showed a significant increase in their paw withdrawal threshold weight compared to those in the PLA and control groups throughout the follow-up observation period after surgery (Figure 8). The von Frey withdrawal threshold weight was 3.60 ± 0.49, 1.80 ± 0.75, and 0.57 ± 0.37 g for the PLA/DHA, PLA, and control groups, respectively, on day 21.

**Figure 9:**
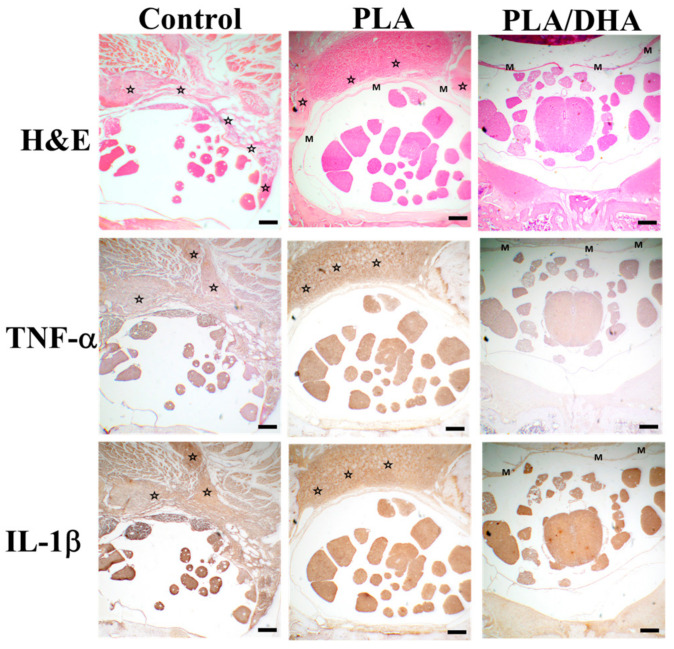
The hematoxylin and eosin (H&E) and immunohistochemical staining of tumor necrosis factor-α (TNF-α) and interleukin-1β (IL-1β) at the lesion site. The animals were subjected to spinal surgery (laminectomy) without treatment (control) or with treatment by implanting a PLA or a PLA/DHA core–shell fibrous membrane (CSFM) post-surgery. Bar = 200 μm. ☆: adhesion or inflammatory tissue. M: fibrous membrane.

The hematoxylin and eosin (H&E) staining of tissue sections at the lesion site after treatment with the PLA or PLA/DHA CSFM is displayed in Figure 9. Severe adhesions developed in the epidural space and surrounding granulation tissues were evident for the untreated control group. Some adhesions were also observed for the PLA group. However, there was no discernible adhesion at the lesion site in the PLA/DHA group. To illustrate the anti-inflammatory characteristic of the DHA/PLA CSFM, the immunohistochemical (IHC) analysis of the key proinflammatory cytokines TNF-α and IL-1 was compared among the different groups. The IHC analysis revealed the expression of TNF-α and IL-1β in the adhesion tissue for both the PLA and control groups. In contrast, the epidural space in the DHA/PLA group did not exhibit any discernible inflammatory tissue when stained for the expression of TNF-α and IL-1.

## 3. Discussion

Post-operative adhesion formation is a common and serious complication after surgery. Using biomaterials as implants to prevent epidural adhesion after lumbar laminectomy has become a primary strategy. Adhesion prevention after spinal surgery can be provided by membrane-type synthetic polymeric materials like poly(lactic-co-glycolic) acid, expanded tetrafluoroethylene, polytetrafluoroethylene, and polyglycolic acid, which can act like a physical barrier to separate the dura mater from the scar tissue [32].

The formation of epidural scar tissue following lumbar surgery might lead to some complications and recurring symptoms, such as radiation or lower back pain, which leads to FBSS. The major underlying cause of these symptoms originates from the extension of the scar tissue with tethered nerve root or dura mater after surgical intervention. Therefore, the implantation of a biodegradable CSFM functioning as a physical barrier was exploited in this study to reduce fibrosis and adhesion formation between the dura mater and surrounding tissues following laminectomy, which has become a primary strategy to prevent epidural adhesion after surgery [33]. Furthermore, surgery-induced inflammatory response contributes to the severity of the scar tissue extending into the neural canal and adhering to the dura mater, which is essential for developing FBSS. Applying a chemical compound at the surgical site following surgery to reduce the inflammatory response is a feasible clinical approach to reduce post-surgical adhesion formation [34]. Considering these needs, a biodegradable PLA-based CSFM was, thus, developed in this study by co-axial electrospinning with infused DHA in the core region of the electrospun fibers, as DHA is considered a potent natural anti-inflammatory compound in pre-clinical and clinical studies [35]. The sustained release of DHA from the PLA/DHA CSFM is expected to provide extended efficacy in managing post-surgical inflammation after lumbar laminectomy (Figure 4). The drug release profile of DHA from PLA/DHA can be explained by simple diffusion-controlled dissolution models, in which the shell layer acts as a protective layer for the drug in the core of the CSFM [36,37]. The loading of DHA in the CSFM can influence the drug release kinetics. A higher DHA mass ratio in the membrane is expected to provide a faster DHA release rate and more DHA in the solution [36,38]. The core–shell structure may also influence the DHA release rate. A CSFM with a thinner shell is expected to show a higher DHA release rate than a thick-shell CSFM where less diffusion length is required for the diffusion of DHA through the shell compartment [39]. The mechanism underlying how the PLA/DHA CSFM prevents adhesion following spinal surgery and reduces neurological discomfort was confirmed from an in vitro study with fibroblasts responsible for adhesion formation. The PLA/DHA CSFM can effectively prevent fibroblast penetration (Figure 5) and reduce the expression of the focal adhesion protein vinculin (Figure 6). This was confirmed in vivo by behavioral data, where sensory impairment was observed in the animals of the control group while the sensory defect was recovered after PLA or PLA/DHA implantation, with the latter showing more significant improvement compared with the former (Figure 8).

We consider the long-term degradation of the PLA/DHA CSFM in vivo. PLA is the most commonly used biodegradable polymer in clinical applications due to its favorable biocompatibility and safe degradation products. It has been approved by the US Food and Drug Administration (FDA) for clinical use. PLA can be completely degraded in the human body, with the primary mechanism being hydrolysis of the ester-bond backbone [40]. The degradation products are lactic acid or carbon dioxide and water, which will be eliminated from the body after being metabolized intracellularly or excreted through urine and breath [41]. A medical implant made from PLA can have high mechanical properties. However, the high molecular weight, hydrophobicity, and high crystallinity of PLA usually lead to a slower degradation rate in vivo, and complete degradation may take up to 2~3 years. The median half-life of this polymer in vivo can be as long as 30 weeks; however, this can be lengthened or shortened by blending, copolymerization, compounding, or surface modification to accelerate the degradation rates of PLA implants to address clinical needs [42]. The half-life of PLA microspheres injected intramuscularly into rats is around 34 weeks [43]. In another study, PLA sutures lost 50% of their weight after ~14 weeks [44]. Upon tissue implantation, the PLA polymer can elicit a foreign body reaction by coating with phagocytic cells and a fibrous capsule. However, adverse reactions or foreign body responses to PLA are infrequent [45]. In the enormous body of clinical cases reporting the use of PLA medical devices, few patients were reported to have allergic responses, and only limited reports described the adverse effects of PLA in patients [46]. PLA orthopedic implant complications may occur with the elicitation of foreign body reactions due to the physical damage to the device, which can be addressed by removing device fragments [47]. As breakage or wearing down of a medical implant can induce foreign body reactions and inflammation, tuning the mechanical properties of the PLA implant is a prerequisite for a successful implant. This is not a concern for the application of the PLA/DHA CSFM given its flexible membrane-like structure.

Another factor related to FBSS that needs to be considered is inflammation-related arachnoiditis around the surgical site. Previous studies have demonstrated that the severity of the clinical symptoms of FBSS could be related to inflammation-related arachnoiditis [8,48]. Numerous reports have shown a direct association between the inflammatory response, the development of fibrosis, and the formation of epidural adhesions [49,50]. In general, this implies that inflammation is among the most significant pathophysiological mechanisms of FBSS. Following spinal surgery, a local inflammatory response typically occurs during the initial period, characterized by processes like hemostasis and coagulation, and the release of chemokines such as phospholipase A2. This, in turn, leads to the accumulation of macrophages, fibroblasts, mast cells, and endothelial cells.

Inflammation is affected by long-chain fatty acids through diverse mechanisms, such as modulation of inflammatory cell function and the inflammation process. DHA, a member of n-3 polyunsaturated fatty acids, is rich in the central nervous system and possesses substantial anti-inflammatory properties [51]. DHA can reduce the inflammatory response by inhibiting endotoxin-induced TNF-α production by cultured monocytes [52]. DHA demonstrates potent anti-inflammatory properties by targeting multiple pathways, notably through the inhibition of nuclear factor kappa-light-chain-enhancer of activated B cell (NFκB) activity. By preventing NFκB nuclear translocation, DHA significantly reduces the expression of key pro-inflammatory cytokines, including IL-1β, IL-6, and TNF-α. Our data revealed a 40–60% reduction in these cytokines in vitro, highlighting DHA’s effectiveness in mitigating inflammation [53,54]. Additionally, DHA serves as a precursor for specialized pro-resolving mediators (SPMs), particularly D-series resolvins (RvD1 and RvD2), which actively terminate inflammatory responses rather than merely suppress them [55,56]. Several clinical and experimental studies have demonstrated that DHA may be a novel and highly efficacious agent to minimize the severity of adhesive disease [22,23,24,25]. A recent study also showed that DHA could largely abolish fibroblast activation and inflammatory cell accumulation [26]. DHA treatment also results in dramatic reductions in the levels of adhesion markers in fibroblasts to a below-normal level compared to untreated fibroblasts in vitro [23]. Previous studies demonstrated that DHA regulates fibroblast activity and prevents fibrosis through key mechanisms. It attenuates TGF-β1-induced Smad2/3 phosphorylation, thereby reducing fibroblast activation into myofibroblasts and mitigating pro-fibrotic effects [57,58]. Furthermore, DHA modulates matrix metalloproteinases (MMP-2 and MMP-9) and their inhibitors, thus decreasing the extracellular matrix deposition and collagen overproduction [59,60,61]. In our vitro study, the inflammatory response of fibroblasts was activated by LPS stimulation (Figure 7). Not surprisingly, the proinflammatory cytokines of fibroblasts were significantly upregulated in the presence of LPS stimulation, similar to the inflammatory response following surgical intervention [62]. Only the extracts of PLA/DHA, but not the extracts of PLA, can downregulate the mRNA expression of proinflammatory cytokines in 3T3 cells (Figure 7). This suggests that DHA released from the PLA/DHA CSFM can retain its ability to reduce inflammation in vitro and is expected to induce less inflammatory response from the surrounding tissues in vivo, which are prone to post-surgical adhesion formation.

The von Frey test developed by the physiologist Maximilian von Frey is a method for evaluating mechanical allodynia in mice [63]. It can be used to assess pains from intervertebral disk degeneration in rats [64], chronically injured spinal cord [65], and spontaneous pain-like behaviors in murine models of postsurgical pain [66]. It was used successfully to assess neuroinflammation in the spinal dorsal horn, where severe nerve injury-induced neuropathic pain arises [67]. Excessive epidural fibrosis and scarring at the operation site can lead to severe adhesion to the dura mater and induce stretching or radicular pressure on the nerve roots and dorsal root ganglia, leading to mechanical radicular pain [68]. Therefore, we assessed whether PLA/DHA can successfully alleviate neuropathic pain-like behaviors in the treated rats by conducting the von Frey filament test before surgery (day 0) and at different time points post-surgery. The mechanical thresholds of the mice in all groups were immediately reduced on day 1 post-surgery. However, the control and PLA groups showed more drastic changes compared with the PLA/DHA group (Figure 8). The mechanical threshold weight represents the sensitivity of the mice to an innocuous mechanical stimulus, with a lower threshold weight observed in the tested animals representing higher sensitivity to the mechanical stimulus. This mechanical hypersensitivity is one of the most common symptoms in neuropathic pain patients. The mechanical threshold increased with time post-surgery but showed distinctive features among the groups (Figure 8). Both the PLA and control groups showed a progressive increase with time. The increase was faster for the PLA group, but there was no significant difference throughout the observation period (Figure 8). In contrast, the threshold weight immediately increased to ~4 g on day 2 for the PLA/DHA group, and this value was maintained throughout the observation period to day 21. Such a significant increase in the threshold weight based on the von Frey test indicates that PLA/DHA has successfully reduced the mechanical hypersensitivity of the mice from post-surgical adhesion.

The histological analysis revealed that the PLA/DHA CSFM can reduce the expression of IL-1β and TNF-α in the rats receiving spinal surgery (Figure 9). This result is consistent with previous studies, which demonstrated that DHA could mitigate the neuro-inflammatory response [69,70]. The expression of IL-1β and TNF-α around the lesion site in the PLA/DHA-treated group was lower than that found in the PLA-treated group on day 21. This indicates that sustained release of DHA from the PLA/DHA CSFM can substantially hinder the inflammatory response by diminishing the expression levels of IL-1β and TNF-α proinflammatory marker proteins.

There has been a surge of research attempting to characterize the role that biodegradable materials play in drug delivery. Local delivery of drugs, such as ibuprofen, lidocaine, mitomycin-C, and steroids, at the surgical site has been provoked to reduce epidural fibrosis after spinal laminectomy [71,72,73,74]. However, high concentrations of these chemical compounds might be cytotoxic to local tissues or the nervous system. DHA is a natural compound with protective effects in the neural system at a single bolus dose [75,76]. Neuroprotectin D1 derived from DHA protects human retinal pigment epithelial cells from oxidative stress and modulates neural cell survival [77,78]. Previous studies also showed that neuroprotectin D1 can effectively reduce neuropathic pain after major surgeries [79]. Subsequently, our designed biomaterial not only meets the anti-inflammatory needs but may also provide an anesthetic effect on the acute response after spinal surgery.

## 4. Materials and Methods

### 4.1. Materials

Dulbecco’s modified Eagle’s medium (DMEM), docosahexaenoic acid (DHA), poly(L-lactide-co-D,L-lactide) (PLA) (L-lactide/D,L-lactide = 70:30, intrinsic viscosity = 2.4 dL/g), actin cytoskeleton/focal adhesion staining kit (FAK100), (3-[4,5-dimethylthiazol-2-yl]-2,5-diphenyl tetrazolium bromide (MTT), and dichloromethane (DCM) were purchased from Sigma-Aldrich (St. Louis, MO, USA). Antibiotics, fetal bovine serum (FBS), and trypsin-EDTA were acquired from Life Technologies (Carlsbad, CA, USA).

### 4.2. Preparation of Core-Shell Fibrous Membrane (CSFMs)

The electrospun core–shell fibrous membranes (CSFMs) were prepared using a co-axial spinneret and a high-voltage power supply providing 25 kV voltages for electrospinning. For the PLA/DHA CSFM, the shell spinning solution contained 10%(*w/v*) PLA prepared in DCM, and the core spinning solution contained 6.54 μg/mL DHA prepared in absolute ethanol. The core and shell spinning solutions were separately delivered by syringe pumps to the spinneret at 0.3 and 1 mL/h. The spinneret was placed horizontally on a rotating drum collector, and core–shell fibers were collected by the grounded collector covered with an aluminum foil. The collector was placed 15 cm from the spinneret and rotated at 2600 rpm. The PLA CSFM was prepared similarly but without adding DHA in the core solution.

### 4.3. Characterization of Core-Shell Fibrous Membranes (CSFMs)

Scanning electron microscopy (SEM) was used to analyze the surface features of the CSFMs. The samples were first coated with a thin gold layer using a sputter coater before being examined at 5 kV with a JEOL JSM-7500F field-emission scanning electron microscope (FE-SEM) (JEOL Ltd., Tokyo, Japan). The average fiber diameter was determined using the ImageJ software (ImageJ 1.53j java 1.8.0_112 (64-bit) version) by randomly choosing 100 fibers from 10 SEM images. To reveal the core–shell structure, each membrane was soaked in distilled water for 3 days and immersed in liquid nitrogen. The frozen membrane was broken from the center to observe its cross section with a JEOL JSM-7500F FE-SEM after coating with gold. For X-ray diffraction (XRD) analysis, a Siemens D5005 X-ray diffractometer (Siemens AG, Munich, Germany) was used by scanning from a 2θ value from 10° to 40° and at a scanning rate of 0.6°/min. Thermogravimetric analysis (TGA) was conducted with 10 mg of each sample in a standard aluminum pan with a Q50 TGA machine from TA Instruments (New Castle, DE, USA). The analysis was conducted from room temperature to 700 °C under a nitrogen atmosphere at a 10 °C/min heating rate. A sessile drop method was employed to measure the water contact angle with an FTA-125 contact angle/surface tension machine from First Ten Angstroms (Portsmouth, VA, USA). The release profile of DHA from the CSFM was assessed by immersing ~10 mg membrane pieces in 1 mL of phosphate-buffered saline (PBS) at pH of 7.4 and 37 °C. At predetermined time points during the 21-day experimental period, the PBS was removed completely for analysis of DHA concentrations, after which 1 mL of fresh PBS was added to continue the drug release experiments. The concentration of DHA in the PBS was quantified using a DHA ELISA kit (American Research Products, Waltham, MA, USA) following the manufacturer’s suggested protocols.

### 4.4. Fibroblast Culture

NIH/3T3 mouse embryonic fibroblasts (BCRC No. 60008) were obtained from the Bioresource Collection and Research Center (BCRC, Hsinchu, Taiwan). NIH/3T3 mouse embryonic fibroblasts were cultured in T-75 flasks with 10 mL of DMEM at 37 °C and 5% CO_2_. After 48 h, the cells were washed with PBS twice and treated with 0.05% trypsin-EDTA for 5 min. After adding 1 mL of DMEM and centrifuging for 5 min at 20 °C, the supernatant was removed, and the cell pellet was mixed with fresh DMEM and added to a cell culture plate. The cells were treated with 0.4% trypan blue and counted with a hemocytometer. Cells between passages 5–15 were used for all experiments to ensure consistency in cellular response.

### 4.5. Cell Cytotoxicity

An in vitro cytotoxicity test was conducted following the ISO10993-5 guidelines with a 24 h extraction medium of the CSFMs. The cell viability was quantitatively assessed using the 3-(4,5-dimethylthiazol-2-yl)-2,5-diphenyltetrazolium bromide (MTT) assay. After extracting ~10 mg of the PLA CSFM or PLA/DHA CSFM with 2 mL of 90% DMEM/10% FBS, 1 mL of the extraction medium was used to culture 3T3 fibroblasts in a 24-well cell culture plate for 24 h. The cell culture medium was removed, and cells were incubated with 400 μL of phenol red-free MTT-containing DMEM (0.5 mg/mL) for 4 h. After incubation and removing the medium, 300 μL of dimethyl sulfoxide was added to dissolve the formed formazan purple crystal in 30 min. The solution absorbance was determined using an ELISA reader at 570 nm (OD_570_). The relative cell viability was obtained by dividing the OD_570_ value with the OD_570_ of control, which used 90% DMEM/10% FBS for cell culture.

### 4.6. Barrier Function from Cell Penetration

The barrier function of the CSFMs was assessed using a double-chamber dish system (Transwell cell culture inserts). The setup involved placing a CSFM at the bottom of a cell insert fitted in the upper chamber of the double-chamber dish system. 3T3 fibroblasts in DMEM/2% FBS were seeded in the upper chamber at a density of 2.5 × 10^5^ cells per well, and the lower chamber was filled with DMEM/10% FBS. Fibroblasts penetrated through the membrane driven by an FBS concentration gradient created in the dish system and were collected at the bottom chamber after being incubated for 24 h at 37 °C. The number of penetrated cells in the lower chamber was observed by direct microscopic observation with an inverted microscope and by DNA assays using Hoechst 33258. The control experiments were conducted without using a CSFM.

### 4.7. Fluorescence Staining of Vinculin and Cytoskeleton

To determine cell attachment on the CSFMs, a 1.5 cm diameter circular-shaped PLA or PLA/DHA CSFM was sterilized in a UV Box (100 μJ/cm^2^) for 4 h. The membrane was placed in a well of a 24-well cell culture plate and covered with an O-ring. A cell suspension of 3T3 fibroblasts (1 ×10^4^ cell/200 μL) was added to the center of the membrane and cultured with 90%DMEM/10% FBS at 37 °C and 5% CO_2_ for 24 h. The focal adhesion and cytoskeletal arrangement of attached 3T3 cells on the membrane surface was visualized using a focal adhesion kit FAK100 to stain the actin cytoskeleton and the focal adhesion protein vinculin. The cells were washed twice with PBS and fixed with 4% paraformaldehyde for 20 min and permeabilized with 0.1% Triton X-100 for 10 min at room temperature. The cytoskeletal distribution was examined by staining F-actin with tetramethylrhodamine (TRITC)-conjugated phalloidin for 30 min. The vinculin staining was carried out by incubating with mouse anti-vinculin primary antibody and fluorescein isothiocyanate (FITC)-AffiniPure goat anti-mouse IgG secondary antibody for 30 min each. The nuclei were counter-stained with 4′,6-diamidino-2-phenylindole (DAPI) for 3 min. The fluorescence-stained cells were visualized using a confocal laser scanning microscope (Zeiss LSM 510 Meta). The actin cytoskeleton, vinculin, and nuclei were stained red, green, and blue, respectively. 3T3 cells cultured on tissue culture polystyrene (TCPS) surface were used for comparison.

### 4.8. Expression of Proinflammatory Cytokine Genes by Reverse Transcription Polymerase Chain Reaction (RT-PCR)

To verify the expression of genes indicative of an inflammatory response, 3T3 cells seeded in 24-well culture plates were subjected to lipopolysaccharide (LPS) stimulation by culturing in a cell culture medium containing 1 μg/mL LPS at 37 °C and 5% CO_2_ for 16 h. As a major component of the Gram-negative bacterial cell wall, LPS can lead to an acute inflammatory response by triggering the release of proinflammatory cytokines in fibroblasts. To investigate the mitigation of inflammation by the PLA or PLA/DHA CSFM, the membrane was soaked with the cell culture medium for 24 h, and the extraction medium was used to culture LPS-stimulated cells. The cells without LPS stimulation and with LPS stimulation alone were used as the negative and positive controls. The mRNA was extracted from the fibroblasts using Trizol, and cDNA was synthesized using a Total RNA Isolation Kit and Maxime RT PreMix Kit following the standard protocols. The expression of proinflammatory cytokine genes, including interleukin-1 (IL-1), interleukin-6 (IL-6), and tumor necrosis factor-alpha (TNF-α), was analyzed using β-actin as a housekeeping gene.

### 4.9. Animal Study

All animal experimental procedures were approved by the Institutional Animal Care and Use Committee (IACUC) of Chang Gung University (IACUC approval number CGU106-028). Lumbar laminectomy was performed on adult female Sprague Dawley (SD) rats (225–249 g) under deep isoflurane anesthesia, confirmed by the absence of paw or corneal reflexes. The procedure began with shaving and disinfecting the rat’s back using povidone-iodine. A dorsal midline incision exposed the back skin and superficial muscles. The L2–L4 laminae were revealed, and a complete laminectomy was executed at L3. A 5 mm longitudinal incision in the dura mater, made with a No. 15 scalpel, resulted in observable cerebrospinal fluid leakage. For the control group, muscle and skin layers were closed sequentially. In the experimental group, the PLA or PLA/DHA CSFM was applied to the lesion site before the layered wound closure. Post surgery, the rats received subcutaneous buprenorphine for pain management and were placed in warm cages for recovery from anesthesia.

For the mechanical allodynia test, a set of ascending, calibrated von Frey filaments (North Coast Medical Inc., Morgan Hill, CA, USA) was used to measure the mechanical response threshold of escape behavior, beginning with the smallest filament (size 1.65, 0.008 g bending force). Von Frey filaments were used to measure the paw withdrawal threshold in the test animals in response to the mechanical stimulus. The type of rat being tested was unknown to the experimenter, and the testing was performed blindly. The filaments were applied to the underside of the fifth toe on the left plantar surface of the hind paw until six consecutive positive or negative responses were noted. The lowest filament’s weight in grams causing a positive response served as the withdrawal threshold.

For histological analysis, the lumbar vertebrae were separated, fixed in 4% paraformaldehyde for 7 days, and decalcified in 30% formic acid for 2 weeks after the rats were sacrificed 4 weeks post-surgery. Following standard protocols, the specimens were sectioned into 4 μm slices and subjected to hematoxylin and eosin (H&E) staining and immunohistochemical (IHC) staining of IL-1β and TNF-α. Under a stereoscopic microscope, the inflammatory response and adhesion formation were examined.

### 4.10. Statistical Analysis

The data were expressed as mean ± standard deviation (SD), and a one-way analysis of variance (ANOVA) was used for statistical analysis, with *p* < 0.05 considered to be significant.

## 5. Conclusions

Aiming to prevent epidural adhesion after laminectomy, we successfully prepared a PLA/DHA CSFM in this study via co-axial electrospinning by infusing DHA in the core of the micro-sized fibers for controlled drug delivery. The membrane can physically block cell penetration, reduce the protein expression of the focal adhesion marker protein vinculin, and reduce the gene expression of proinflammatory cytokines by fibroblasts in vitro. When tested as an anti-adhesion implant in a laminectomy rat model, the PLA/DHA CSFM demonstrates anti-inflammatory and anti-adhesion effects as revealed from behavioral assessment and histological analysis. Consequently, the introduction of an electrospun CSFM designed to sustain anti-inflammatory effects could have a significant influence on mitigating FBSS following spinal surgery by inhibiting the formation of epidural fibrosis and arachnoiditis. The potential clinical translation of the PLA/DHA CSFM for managing FBSS is expected, as well as its potential utility in abdominal and muscle/tendon surgery in the future.

## Data Availability

The data presented in this study are available from the corresponding author upon request.
